# Health in Factories

**Published:** 1919-12-13

**Authors:** 


### HEALTH IN FACTORIES.

Coming at a time when increased production and the contentment of the working classes are of such vital importance to the nation's post-war recuperation, the report* for 1918 issued by the Chief Inspector of Factories is a more than usually valuable and interesting document. Not only does it summarise most of the industrial health problems solved during the period of hostilities, but it provides also many instructive details for future methods of improvement. Previously one has been accustomed to a somewhat dull statistical record of these things. Mostly a record which conveyed little to the average medical reader and still less to the layman. In this issue, however, we read eight chapters written by experts on the matters in hand and describing clearly the direct and indirect bearing of such important considerations as hours of labour, labour-saving devices, accident prevention, electricity in industry, industrial poisoning, etc. They are dealt with not from the standpoint of figures alone but clear arguments, excellent descriptions—and practical applications are given with each. Throughout there is evidence that men fully qualified to judge have worked upon these vital problems and, although in the past similar expertness has doubtless been in reality employed, yet this is the first of these publications which has of itself proved such presence in the form of its presentation and the wealth of information it provides. We trust that the practice may be continued in future years.

In so short a space it is impossible to make any extensive extracts, but there are a few points to which, in illustration, attention may well be drawn. On the vexed question of reduced hours resulting in increased output, it is to be noted that case after case is provided, taken from a great variety of trades, where this sequence is obtained, and where—which is even more important—there was also great reduction in lost time and sickness. The introduction of the one-break day and the abolition of before breakfast work, particularly with the women workers, are proving definitely advantageous, and the conclusion inevitably drawn from this section is that hours can be very considerably reduced without either reducing output or increasing the cost of production. In close association comes the value of labour-saving appliances—not in major machinery alone but also in improved efficiency of the numerous articles which must be handled daily. One of the main effects of increased female employment has been that more attention was paid to adjusting of tables and benches to the height of workers, the provision of lighter tools—and a host of simple but important improvements. The employer of labour may indeed learn many lessons from this part of the report, profitable not only to his workers, but to himself.

Of the purely scientific researches described none are more interesting than the various industrial poisonings, the incidence of which has been so successfully countered in recent times. The presence, pathology, and administrative causes of these conditions have been partially known for many years, but the valuable chapter written by Dr. T. M. Legge takes us further than anything read before—as far, in fact, as almost complete prevention, instead of the old-time methods of merely reducing the situation to one of smouldering menace. With regard to accident prevention we are glad to see such whole-hearted support of a view that was already becoming widely realised. No longer does the report give the impression that reduction in number of injuries is to be effected only by ever multiplying the mechanical safety devices. The psychology of the workers is brought out as an almost paramount factor. "A very large number of accidents occur through the neglect of simple precautions on the part of the workers, which is often due to the want of training, defective organisation, or absence of discipline in the factory." Before a definite improvement occurs, a joint effort must be made by employers and workers in each individual instance, and the system of properly constituted safety committees is greatly to be commended.

In a similar manner the reader encounters instance after instance of that type of change or precaution, the very simplicity of which has caused it to be overlooked in the recent years, and the point we would urge is that, interesting though the report is to a profession intimately connected with the public health and welfare, yet the country has before it a document which should be read by a far greater circle. There is no one in any position of industrial control who will not find his attention held by the improvements and reforms suggested, and who, in acting conscientiously upon the very sound principles brought forward, can fail materially to benefit the employer, the worker, and his country.

* H.M. Stationery Office Price 9d. net.

